# Editorial: Sensory systems of aquatic animals

**DOI:** 10.3389/fphys.2024.1432649

**Published:** 2024-05-23

**Authors:** Jeremy M. Sullivan, Laure Bonnaud-Ponticelli, Anna Di Cosmo

**Affiliations:** ^1^ Department of Neurology, Johns Hopkins University School of Medicine, Baltimore, MD, United States; ^2^ UMR Biologie des Organismes et Ecosystèmes Aquatiques, Muséum National d'Histoire Naturelle, CNRS 8067, Sorbonne Université, Paris, France; ^3^ Department of Biology, University of Naples Federico II, Naples, Italy

**Keywords:** aquatic animals, audition, olfaction, sensory systems, TRP channels, vision

The external world is filled with a myriad of sensory stimuli (e.g., mechanical, chemical, thermal), the detection and interpretation of which are key to an animal’s survival. It is imperative, for example, that animals be able to quickly and accurately distinguish the odours of prospective food items, potential mates, and nearby predators. In animals, external stimuli are perceived principally by classes of receptor cells specialized for the detection of specific sensory stimuli (e.g., visual, auditory, olfactory, gustatory, somatosensory). Each receptor cell class is innervated by a dedicated population of sensory neurons that transmit information about the stimulus to the central nervous system for processing and interpretation. In addition, many cells, both neuronal and non-neuronal, express specialized ion channels that respond to changes in environmental stimuli (e.g., thermosensitive TRP channels).

Aquatic animals inhabit a diverse array of environments, from freshwater streams, to estuaries, to the deep ocean. The distinct features of these environments have shaped the evolution of the sensory systems of aquatic animals, resulting in specializations suited to each habitat. Elucidation of the ways in which aquatic species sense and interpret their environments contributes not only to an increased understanding of their biology, but also plays important roles in assessing the impacts of ongoing changes in aquatic environments, such as increasing levels of anthropogenic noise pollution and global warming. In this special issue, we present four articles examining different sensory systems of aquatic vertebrate and invertebrate animals. These articles provide new insights into the auditory (Rojas et al.), olfactory (Ferrando et al.), and visual (Gao et al.) systems of diverse aquatic species, as well as into TRP channel biology (Wang et al.).

Here, Rojas and colleagues present the first ultrastructural description of the auditory receptor organ, the organ of Corti, of the harbor seal (*Phoca vitulina*). Phocid seals, such as harbor seals, exhibit several specializations of the outer and inner ear. Though previous functional studies have characterized the hearing range of harbor seals, supporting anatomical studies have been lacking. Utilizing scanning electron microscopy, Rojas and colleagues mapped the distribution of receptor cell populations along the length of the organ of Corti. These findings will form the basis for future work establishing a tonotopic map for the harbor seal cochlea. Importantly, an understanding of sound frequency mapping within the organ of Corti will be key for future pathological studies examining effects of human-derived noise on hearing loss in phocid seals.

The olfactory organs of aquatic animals exhibit a high degree of specialization based on lifestyle, diet, and habitat. In their article, Ferrando and colleagues present a detailed morphological analysis of the olfactory organs of 14 species of cartilaginous fish (Chondrichthyes). Specifically, the authors focussed on secondary folds, present along the surface of the olfactory organs of these fishes, which expand the surface area of the olfactory epithelium. Strikingly, individual species showed marked variability in the increase in olfactory epithelial surface area provided by the secondary folds, ranging from 70% to 495%. These findings will enable future work examining the correlation between secondary fold complexity, olfactory receptor neuron innervation, and olfactory biology across chondrichthyan species.

In their review, Gao and colleagues present an overview of electroretinography (ERG) studies of the visual systems of aquatic animals. ERG methods assess electrical potential changes in retinal cells in response to light and have been instrumental in elucidating the visual sensitivities of both aquatic vertebrates and invertebrates. In particular, the authors focus on the key findings of ERG studies of fishes, crustaceans, and molluscs. Variability between the visual sensitivities of individual aquatic animal species is discussed in the context of the animals’ habitats and lifestyles. The impact of environmental factors, such as temperature, on visual function are also outlined. Finally, the authors discuss the importance of incorporating the findings of anatomical studies, as well as refined analytical and pharmacological approaches, to enable the discrimination of individual retinal cell populations in ERG studies.

The TRP channel superfamily comprises a diverse array of polymodal channels activated by a variety of chemical and physical stimuli. Here, Wang and colleagues utilized transcriptomic data sets to present the first characterization of the TRP channel superfamily members of the echinoderm *Apostichopus japonicus*. These analyses led to the identification of 54 TRP channels, belonging principally to six subfamilies. As *A. japonicus* is poikilothermic, the authors also characterized changes in the expression levels of several TRP channels, including putative thermosensitive TRP channels, under conditions of heat stress. These findings will contribute to our understanding of the effects of climate change-mediated temperature fluctuations on echinoderm biology.

Together, these articles provide valuable and timely insights into several key aspects of the sensory biology of aquatic animals. Importantly, many aspects of this work will have implications for the management of anthropogenic impacts on the habitats of these species. We, the editors, would like to extend sincere thanks to all the authors for their contributions to this special issue.

